# Mechanisms and Neuroprotective Activities of Stigmasterol Against Oxidative Stress-Induced Neuronal Cell Death *via* Sirtuin Family

**DOI:** 10.3389/fnut.2021.648995

**Published:** 2021-05-12

**Authors:** Reny Pratiwi, Chanin Nantasenamat, Waralee Ruankham, Wilasinee Suwanjang, Virapong Prachayasittikul, Supaluk Prachayasittikul, Kamonrat Phopin

**Affiliations:** ^1^Center of Data Mining and Biomedical Informatics, Faculty of Medical Technology, Mahidol University, Bangkok, Thailand; ^2^Department of Medical Laboratory Technology, Faculty of Health Science, Setia Budi University, Surakarta, Indonesia; ^3^Center for Research and Innovation, Faculty of Medical Technology, Mahidol University, Bangkok, Thailand; ^4^Department of Clinical Microbiology and Applied Technology, Faculty of Medical Technology, Mahidol University, Bangkok, Thailand

**Keywords:** stigmasterol, neuroprotection, oxidative stress, apoptosis, antioxidant, SIRT1

## Abstract

**Background:** Accumulating studies have confirmed that oxidative stress leads to the death of neuronal cells and is associated with the progression of neurodegenerative diseases, including Alzheimer's disease (AD). Despite the compelling evidence, there is a drawback to the use of the antioxidant approach for AD treatment, partly due to limited blood-brain barrier (BBB) permeability. Phytosterol is known to exhibit BBB penetration and exerts various bioactivities such as antioxidant and anticancer effects, and displays a potential treatment for dyslipidemia, cardiovascular disease, and dementia.

**Objective:** In this study, the protective effects of stigmasterol, a phytosterol compound, on cell death induced by hydrogen peroxide (H_2_O_2_) were examined *in vitro* using human neuronal cells (SH-SY5Y cells).

**Methods:** MTT assay, reactive oxygen species measurement, mitochondrial membrane potential assay, apoptotic cell measurement, and protein expression profiles were performed to determine the neuroprotective properties of stigmasterol.

**Results:** H_2_O_2_ exposure significantly increased the levels of reactive oxygen species (ROS) within the cells thereby inducing apoptosis. On the contrary, pretreatment with stigmasterol maintained ROS levels inside the cells and prevented oxidative stress-induced cell death. It was found that pre-incubation with stigmasterol also facilitated the upregulation of forkhead box O (FoxO) 3a, catalase, and anti-apoptotic protein B-cell lymphoma 2 (Bcl-2) in the neurons. In addition, the expression levels of sirtuin 1 (SIRT1) were also increased while acetylated lysine levels were decreased, indicating that SIRT1 activity was stimulated by stigmasterol, and the result was comparable with the known SIRT1 activator, resveratrol.

**Conclusion:** Taken together, these results suggest that stigmasterol could be potentially useful to alleviate neurodegeneration induced by oxidative stress.

## Introduction

Oxidative stress occurs in many human diseases as a result of the imbalance between high levels of reactive oxygen species (ROS) production and insufficient antioxidant defense resulting in lipid peroxidation, cellular membrane impairment, protein degradation, and DNA damage (1). Because of the high lipid content and the high basal oxygen (O_2_) consumption, the human brain is susceptible to oxidative damage caused by ROS (2, 3). Excessive amounts of ROS induce neuronal cell death and are associated with neurodegenerative diseases, including Alzheimer's disease (AD) (4). The origin of AD is complex and not well-understood, however, the involvement of oxidative stress in the pathogenesis and the progression of AD have been reported (5–7). AD is the most common cause of neurodegenerative diseases and is increasingly recognized as a major public health burden. According to the World Health Organization report in 2020, AD has affected millions of people and has become the leading cause of death worldwide (8). In addition to this burden, current treatments are ineffective and unable to stop the progression of AD. Therefore, the discovery of new therapeutics for AD treatment has become a research priority (9–11).

Over the centuries, nature has been the origin of therapeutic compounds, as it offers an abundant chemical diversity and larger chemical space than synthetic compounds (12, 13). Plant-derived compounds, especially phytosterols have been associated with health benefits. Phytosterols are abundantly found in plant oils (corn, soybean, sunflower, and olive), peanuts, cereals, almonds, avocados, and vegetables, ranging from 50 to 220 mg/100 g, and entering the human body through diet (14, 15). Phytosterols are also found in other parts of medicinal plants i.e., *Spilanthes acmella* Murr. (16–18), *Hydnophytum formicarum* Jack. (19), *Coriandrum sativum* (20), and *Sargassum fusiforme* (21). Phytosterol has been reported to have cholesterol lowering properties and the potential to be used for dyslipidemia treatment (22). Other potential benefits of phytosterol, include anti-cancer (23), antioxidant (15), and anti-atherosclerotic activities (24). Due to their distinct abilities to penetrate BBB and accumulate in the brain, investigation of the physiological role of phytosterol in neurodegenerative disorders has been initiated (14, 25, 26), and a recent report suggested that phytosterol could potentially be used in neurodegenerative treatment (27).

**Figure 1:**
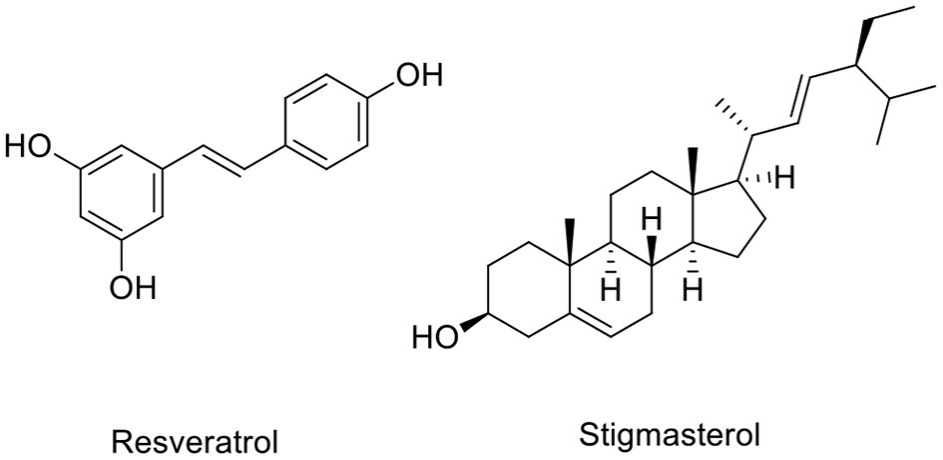
Chemical structures of resveratrol and stigmasterol.

Amongst the diversity of phytosterol compounds, stigmasterol is most commonly found in plants such as vegetables, fruits, and berries, including carrot, dill, parsley, red beet, kiwi fruit, alfalfa seed, and peanut (28). Structurally, stigmasterol is similar to that of cholesterol in mammals, with the distinction of a double bond at the C22 position (Figure 1) (29, 30). Various studies have reported the involvement of stigmasterol in neuroprotection through several mechanisms. Stigmasterol isolated from medicinal plants displayed acetylcholinesterase inhibitory activity *in vitro* (31). This compound also decreased the β-secretase cleavage of amyloid precursor protein (APP) and eventually reduced amyloid β *via* attenuation of β-site APP cleaving enzyme 1 (BACE1) internalization from the surface of the cells into the cell compartments (32). However, the underlying neuroprotective mechanisms of this plant sterol are not well-understood, particularly the pathway controlled by the sirtuin family mechanism.

A recent study has reported the endogenous 24-hydroxycholesterol involved in neuroprotection by modulation of sirtuin 1 (SIRT1) and suggested that different types of sterols correlate with disease stages (33). It has been reported from *in vitro* and *in vivo* studies that the increase of SIRT1 protein reduces AD-like disorder (34). Correspondingly, an *in vitro* study also showed that the decrease of Aβ oligomer formation *via* activation of SIRT1 by either NAD^+^ or resveratrol (Figure 1) leaded to upregulation of the APP metabolism by α-secretase (35). Moreover, overexpression of SIRT1 protected SH-SY5Y cells from toxicity-induced cell death (36). Therefore, this study set out to investigate the neuroprotective effects of stigmasterol against H_2_O_2_-induced oxidative stress in SH-SY5Y neuroblastoma cells and explore the possible neuroprotective pathways *via* SIRT1-FoxO3a modulated by stigmasterol.

## Materials and Methods

### Chemicals and Reagents

All chemicals and reagents employed herein are analytical grade. The following compounds and assay kits were acquired from Sigma-Aldrich, Inc.: stigmasterol, resveratrol, trypsin-EDTA (0.25%), hydrogen peroxide (H_2_O_2_), dimethyl sulfoxide (DMSO), 3-(4,5-dimethylthiazol-2-yl)-2,5-diphenyltetrazolium bromide (MTT), and rhodamine 123 (Rho 123) fluorescent dye. Dulbecco's minimum essential medium (DMEM), fetal bovine serum (FBS), and penicillin/streptomycin were obtained from Gibco, a Thermo Fischer Scientific company. Carboxy-DCFDA assay kit (Invitrogen), Muse™ Annexin V and Dead Cell (Merck Millipore, Billerica, MA, USA), and the Bio-Rad Bradford protein assay reagent (Hercules, CA, USA) were also used in this study. The following antibodies were used for the Western blot analysis and purchased from Cell Signaling Technology, Inc.: mouse monoclonal antibody anti-SIRT1 and anti-Bcl-2, rabbit monoclonal antibody anti-β-actin, anti-catalase, anti-FoxO3a, and anti-acetylated-lysine antibodies. Secondary antibodies were anti-mouse IgG horseradish peroxidase (HRP)-linked antibody and anti-rabbit IgG HRP-linked antibody. Amersham Enhanced Chemiluminescence (ECL) Prime Western Blotting Detecting Reagent (GE Healthcare) was used for immunoblotting. Unless otherwise stated, all chemicals and reagents were purchased from Sigma-Aldrich.

### Preparation of Tested Compounds

Stock solutions were prepared as follows, stigmasterol was dissolved in polyethylene glycol (PEG) and resveratrol was diluted in DMSO for indicated concentrations. A 1 μM of the working solution was prepared by diluting the stock solution in DMEM added with 10% of FBS. Resveratrol was used as the reference compound of known SIRT1 activator.

### Cell Culture and Treatment

SH-SY5Y neuroblastoma cells were maintained in complete media containing DMEM enriched with 10% FBS and 1% penicillin and streptomycin mixture at 37°C under 5% CO_2_ and 95% humidified air incubator. The media were changed every 2 days, and the cells were passaged at ~80% of confluence. Cells were pretreated with 1 μM stigmasterol or 1 μM resveratrol for 3 h, and untreated cells as the control. After the pretreatment, culture media containing stigmasterol or resveratrol were replaced with fresh media following by incubation with H_2_O_2_ for an additional 24 h. It has been reported that 400 μM of H_2_O_2_ induced cell death by 34–35% and therefore was applied in this study (37).

### Cell Morphology Assessment

The cells were prepared at a density of 1 × 10^5^ cells/mL in the 6 well plates. Following cell seeding and pretreatment with compounds, culture media were refreshed, followed by H_2_O_2_ exposure for a further 24 h. The cell morphology was evaluated using Olympus inverted microscope at 20× magnification. The images of each treated and untreated cell from multiple independent fields were documented using a digital camera.

### Cell Viability Assay

Cell viability was measured by performing an MTT assay (38). Briefly, in viable cells, MTT is converted to blue formazan product by dehydrogenase enzymes. The cell viability of each group treated with compounds and H_2_O_2_ was calculated as a percentage relative to the cell viability of the control group. SH-SY5Y cells (1 × 10^5^ cells/mL) in a 96 well plate were pretreated for 3 h with stigmasterol or resveratrol prior to incubation with 400 μM H_2_O_2_ for 24 h. Subsequently, MTT solution (5 mg/mL) was added into each well and stored at 37°C for 2–4 h in dark conditions. At the end of the incubation time, the MTT solution was discarded and 0.04 N HCl in isopropanol was added as extraction buffer. Absorbance was determined at a wavelength of 570 nm *via* a microplate reader (Bio-Tek Instruments, Inc., Winooski, VT, USA).

### Reactive Oxygen Species Measurement

The production of intracellular ROS was detected using carboxy-DCFDA, a ROS fluorescent probe. Briefly, non-fluorescent carboxy-DCFDA is oxidized to highly green fluorescent dichlorofluorescein (DCF) in the presence of ROS, which is detected by a fluorescence plate reader. Cells were plated at a concentration of 1 × 10^5^ cells/mL in a 96 well plate and allowed to attach. Subsequently, the cells were treated with stigmasterol or resveratrol at 1 μM for 3 h prior to incubation with 400 μM H_2_O_2_ for 24 h. Untreated cells in complete media with 0.1% DMSO were employed as the control. To remove excess media after incubation, cells were subjected to washing with phosphate-buffered saline (PBS). Furthermore, DCFDA was applied to each well to reach a final concentration of 25 μM in culture media and incubated at 37°C for 30 min in dark conditions. ROS formation was assessed using a microplate reader at an excitation wavelength of 492–495 nm and an emission wavelength of 517–527 nm. The intracellular ROS production levels of treated cells were expressed as a percentage relative to untreated cells.

### Mitochondrial Membrane Potential Assay

In this study, the measurement of mitochondrial membrane potential (MMP) was performed using the mitochondrial-specific fluorescent dye Rho 123 (39, 40). Following the electrochemical gradient, Rho 123 accumulates and remains in the mitochondria in an equivalent amount of the MMP. The reduction of Rho 123 fluorescence signal correlates with the decrease of MMP (40). The SH-SY5Y cells (1 × 10^5^ cells/mL) in a 96 well plate were pretreated with stigmasterol or resveratrol for 3 h followed by incubation with 400 μM H_2_O_2_ for an additional 24 h. At the end of the incubation period, cells were subjected to incubation with 10 μM Rho 123 for ~30 min at 37°C. Next, cells were washed with PBS to remove the excess Rho 123 dye. MMP was measured using a microplate reader at excitation and emission wavelengths of 485 and 528 nm, respectively, and calculated as a percentage relative to the untreated control cells.

### Apoptotic Cell Measurement

The percentage of oxidative stress-induced apoptotic cells was measured using Muse™ Annexin V and Dead Cell assay containing Annexin V and 7-amino-actinomycin D (7-AAD) fluorescent markers. The cells were prepared at a density of 1 × 10^5^ cells/mL in the 6 well plates. After cell seeding and pretreatment with compounds, culture media were refreshed with new media followed by H_2_O_2_ exposure for a further 24 h. At the end of the incubation period, all cells were collected and centrifuged for 5 min at 1,000 RPM, then prepared in 1 mL of complete media. Then, 100 μL of cell suspension was stained for 20 min in dark conditions with 100 μL of fluorescent reagent at room temperature. Annexin-V binds with phosphatidylserine (PS) on the external surface of apoptotic cells, whereas 7-AAD labels the dead cells. At the end of the incubation time, the percentage of living and dead cells was measured using Muse™ Cell Analyzer, Merck Millipore (41).

### Detection of SIRT1, Acetylated Lysine, FoxO3a, Bcl-2, and Catalase in Human Neuronal Cells Treated With Stigmasterol by Western Blot Assay

In this study, Western blot assay was employed to detect SIRT1, acetylated lysine, FoxO3a, Bcl-2, and catalase protein expressions according to the protocol described herein (37, 42). In the cell culture dishes, 1 × 10^5^ cells/mL of SH-SY5Y cells were seeded overnight followed by pretreatment with 1 μM of stigmasterol or resveratrol for 3 h. After the pretreatment, culture media containing a compound were replaced with fresh media followed by incubation with 400 μM H_2_O_2_ for 24 h. Control cells or untreated cells were incubated in media enriched with 10% FBS for 24 h. At the end of the incubation time, the cells were subsequently washed with PBS and extracted in RIPA lysis buffer containing protease inhibitors at 4°C for 20 min. Then, cells collected from the culture dish were subjected to sonication for 10 sec and centrifugation at 10,000 RPM for 20 min at 4°C. The total protein concentration in each sample was measured using the Bradford protein assay.

Furthermore, 25 μg of total protein extracts from each sample was separated by 12% SDS-PAGE and subsequent electrophoretic transfer to PVDF membrane. Afterward, the blocking of the membrane was achieved using 5% skim milk in Tris-buffered saline containing 0.1% Tween-20 (TBST) for 1 h at room temperature followed by three washing intervals for 5 min with TBST. The membrane was subjected to incubation with specific primary antibodies (anti-SIRT1, anti-acetylated lysine, anti-FoxO3a, anti-Bcl-2, and anti-catalase antibodies) at 4°C overnight. The membrane was applied to three washing intervals for 5 min with TBST, incubation for 2 h with HRP-linked secondary antibody, and finally three washing intervals for 5 min with TBST. Protein bands were detected using the ECL reagent. The intensity of each band was analyzed to quantify the levels of protein using the Image Lab software (Bio-Rad). The levels of protein of the H_2_O_2_-treated group were compared with that of cells treated with compounds and H_2_O_2_. Finally, the percentages relative to the protein level of β-actin and the control group were calculated.

### Binding Affinity Calculation of SIRT1 and Stigmasterol

Molecular docking analysis was performed to examine the binding affinity and interaction of stigmasterol and resveratrol against SIRT1. The 2D structures of stigmasterol and resveratrol were prepared with MarvinSketch and transformed into 3D PDB format using built-in MolConverter in JChem Suite (www.chemaxon.com; Chemaxon, Life Sciences, Informatics, Cheminformatics, Budapest, Hungary). An X-ray crystallographic structure of human SIRT1 in complexation with resveratrol and a 7-amino-4-methylcoumarin (AMC)-containing peptide (PDB accession code 5BTR) at a resolution of 3.2 Å was obtained from the RSCB Protein Data Bank (43, 44). Subsequently, resveratrol was used as the reference compound for SIRT1 activation analysis. In preparation for the docking simulation, the protein structure was subjected to the removal of the ligand and water molecules to produce a ligand-free protein structure using PyMol (45). Polar hydrogen atoms and Gasteiger charges were added to the protein, the charges were merged, and non-polar hydrogens and lone pair atoms were removed by using the automated docking tool, AutoDockTools (46, 47).

AutoDock Vina was used for docking simulation to improve scoring functions, efficient optimization, and multi-threading properties (48). Briefly, a grid box size (60 × 60 × 60 points) with a spacing of 0.375 Å between grid points was generated to ensure coverage of all favorable protein binding sites. The x, y, z Cartesian coordinate of the center of the grid box was set to −26.047, 59.839, and 9.038, respectively. Molecular docking was performed with default parameters. The binding energy (kcal/mol) of predicted binding modes was obtained from AutoDock vina calculation and was compared with the reference compound, resveratrol. Binding site interactions were displayed using a 2D interaction diagram generated by the PLIP webserver (49) and BIOVIA, Discovery Studio 4.5 software (50).

### Statistical Analysis

Statistical significances were analyzed by one-way ANOVA followed by Tukey's multiple comparison test using the significant level of *p* < 0.05 carried out by GraphPad Prism version 6.01 for Windows, GraphPad software, La Jolla, California, USA, (Available at http://www.graphpad.com). Results were represented as the mean of three independent experimental values ± S.E.M.

## Results

### Effects of Stigmasterol on Cell Viability

**Figure 2:**
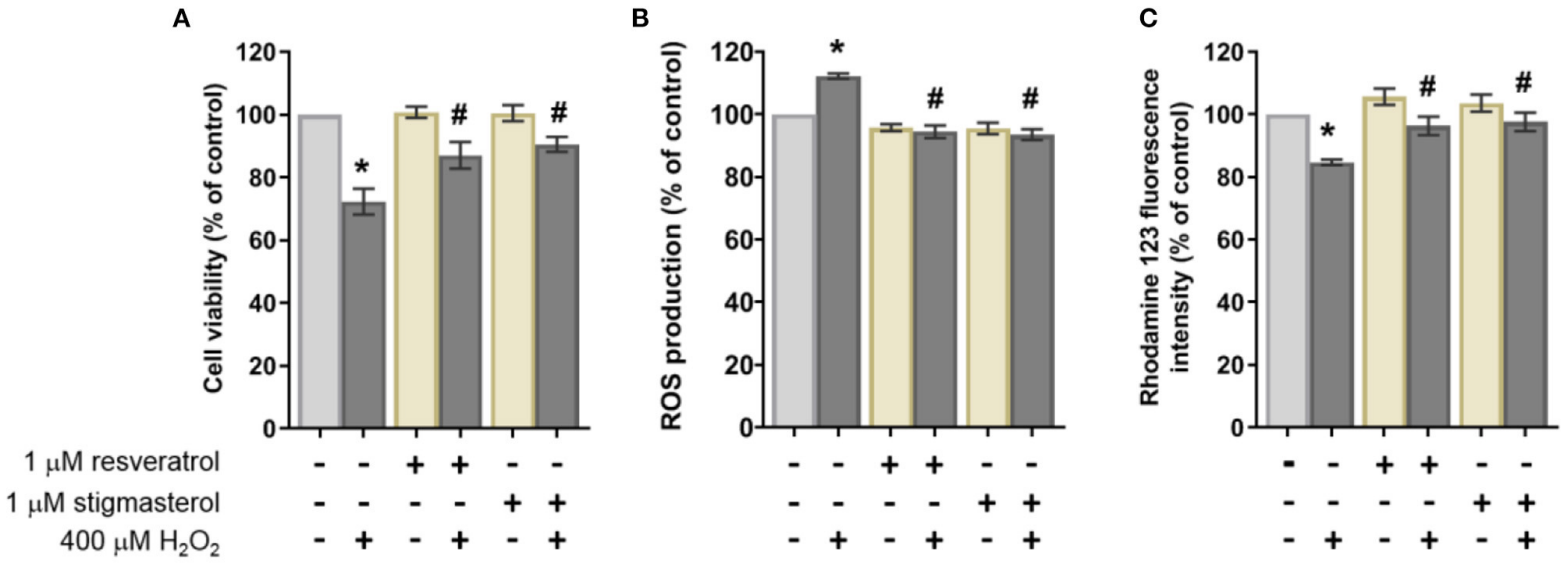
Effects of 1 μM resveratrol and stigmasterol pretreatment for 3 h followed by 400 μM H_2_O_2_ exposure for 24 h of SH-SY5Y cells on the **(A)** cell viability, **(B)** intracellular ROS production, and **(C)** mitochondrial membrane potential (MMP) as measured by the Rhodamine 123 fluorescence intensity (% of control). Data are presented as mean ± S.E.M. of three independent experiments (*p < 0.05 vs. control; ^#^*p* < 0.05 vs. 400 μM H_2_O_2_-treated group).

To determine the effects of stigmasterol on cell viability, the cells were pretreated with each compound for 3 h. After the incubation, 400 μM H_2_O_2_ was added to the culture media for 24 h, followed by cell viability determination. The results revealed that H_2_O_2_ significantly induced neuronal cell death by 28%. On the other hand, the cell survival rate was ~96 ± 5.3% in stigmasterol-treated cells with a 24% recovery of cell viability compared with H_2_O_2_ treatment (Figure 2A).

### Effects of Stigmasterol on H_2_O_2_-Induced Intracellular ROS Production

Intracellular ROS was measured to evaluate the ability of stigmasterol to protect the cells from excessive intracellular ROS production following H_2_O_2_ induction. The ROS levels in the cells were detected using DCFDA fluorescent probe by measuring the intensity of fluorescent compound dichlorofluorescein (DCF) as a product of ROS and probe reaction. The fluorescent signal of pretreated groups was compared with the control group after being exposed to 400 μM H_2_O_2_ for an additional 24 h. The reduction of fluorescent intensity was observed in the cells pretreated with compounds (~20%) as shown in Figure 2B. This observation suggested that stigmasterol significantly attenuated intracellular ROS production. The comparable results were obtained from the group pretreated with resveratrol.

### Effects of Stigmasterol on Mitochondrial Membrane Potential

Reduction of the mitochondrial membrane potential (MMP or ΔΨm) has been associated with mitochondrial dysfunction and apoptosis (51). In this study, the reduction of MMP was observed in the H_2_O_2_-treated cells (84.7 ± 0.9%) compared with the cells pretreated with stigmasterol and resveratrol followed by 24 h incubation with H_2_O_2_ (97.7 ± 2.9% and 96.3 ± 2.9%, respectively) as presented in Figure 2C. It indicated that the compounds can maintain the levels of MMP in the neuroblastoma cell lines.

### Morphological Observation of Stigmasterol-Treated Neuronal Cells by Light Microscope

**Figure 3:**
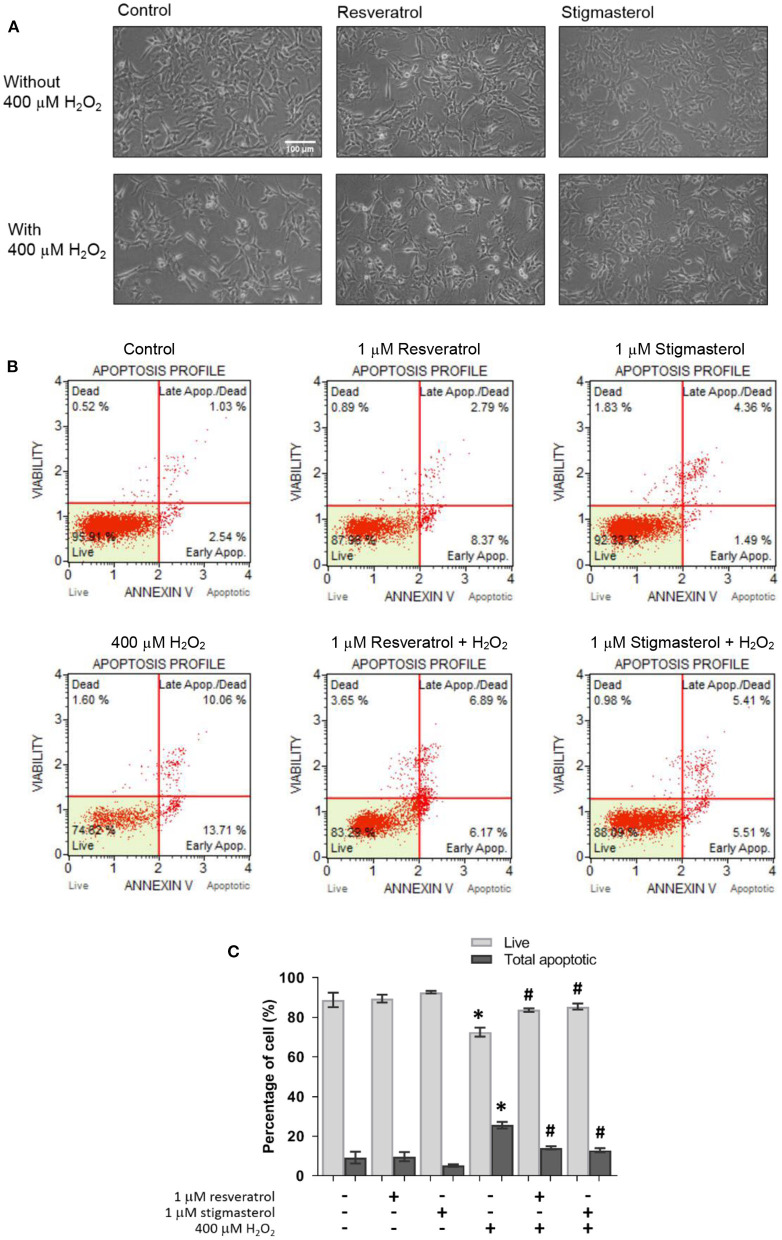
Resveratrol and stigmasterol inhibited apoptosis caused by H_2_O_2_. **(A)** Morphology of SH-SY5Y cells was observed under the inverted-light microscope at 20 × magnification, scale bar 100 μm. **(B)** Representative plots of live and apoptotic cells as measured by flow cytometry on pretreatment of 1 μM resveratrol and stigmasterol exposed to 400 μM H_2_O_2_ for 24 h. **(C)** Bar chart of live and total apoptotic cells. Data are presented as mean ± S.E.M. of three independent experiments (*p < 0.05 vs. control; ^#^*p* < 0.05 vs. H_2_O_2_-treated group).

To monitor the effects of stigmasterol on the morphology of toxicity-induced neuronal cells, the morphological assessment of the stigmasterol-pretreatment cells followed by 400 μM H_2_O_2_ treatment was performed under a light microscope. Under oxidative stress, neuronal cell damage including shrinkage of the cells and a decrease in cell numbers were found in the cells incubated with H_2_O_2_ compared with the control cells (Figure 3A). The data indicated that 1 μM of stigmasterol and resveratrol conserved the cells from H_2_O_2_-induced oxidative damage by maintaining cell intact and cell growth with adequate cell confluence.

### Effects of Stigmasterol on H_2_O_2_-Induced Apoptosis in SH-SY5Y Cells

To determine the protective effects of stigmasterol against cell apoptosis induced by H_2_O_2_, an Annexin V/7-AAD assay was conducted. As presented in Figure 3B, the number of apoptotic cells was significantly increased after H_2_O_2_ treatment. The percentage of apoptotic cells in the H_2_O_2_-treated group was higher (25.5 ± 1.7%) than in the cells pretreated with 1 μM of stigmasterol and resveratrol (12.9 ± 1.0% and 14.0 ± 0.8%, respectively). However, the results also showed no significant difference in apoptotic cells treated only with the compounds compared with the untreated control group (Figure 3C). These findings suggested that stigmasterol protected neuronal cells from H_2_O_2_-stimulated apoptosis.

### Stigmasterol Effects on Anti-apoptotic Protein Expression

**Figure 4:**
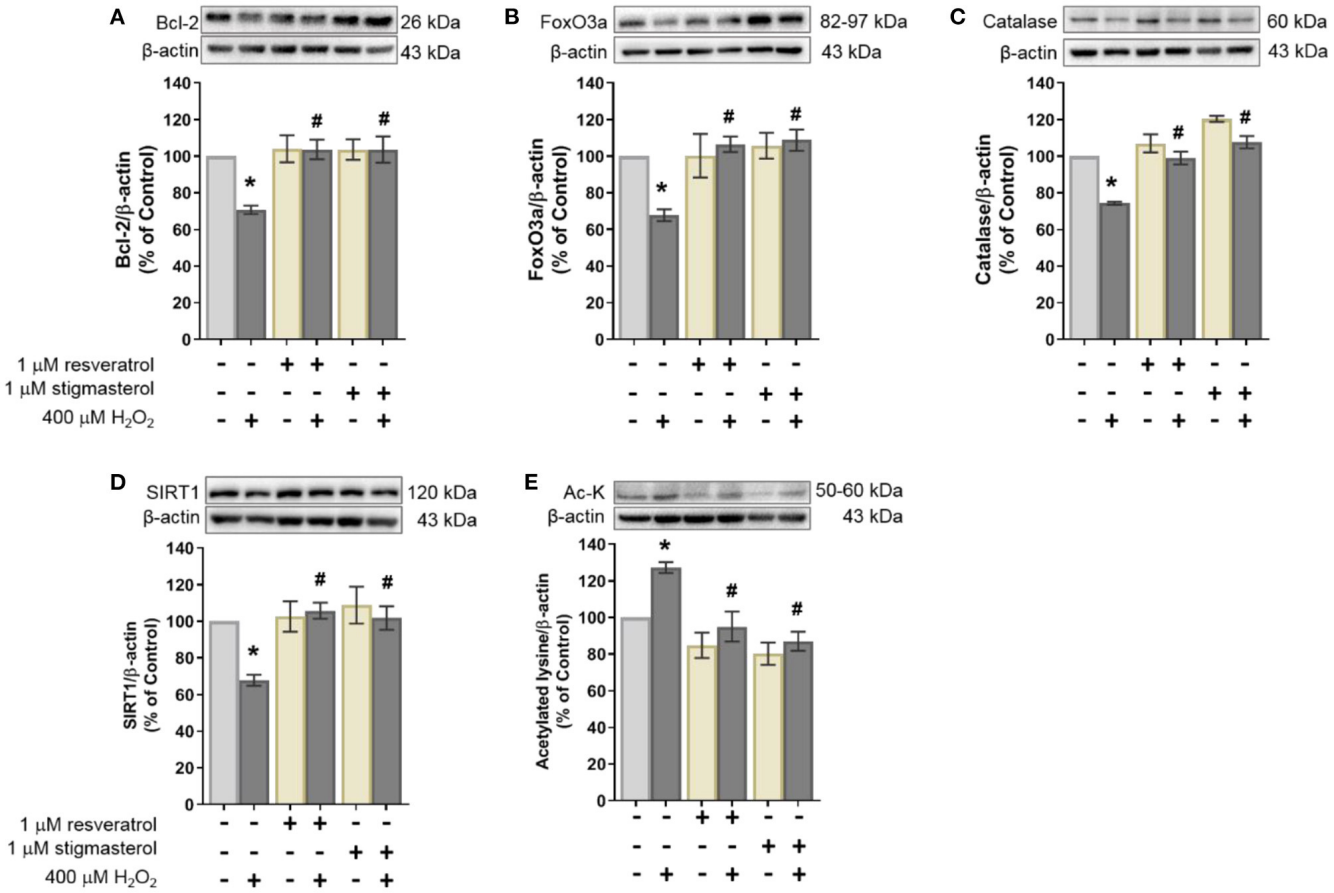
Effects of resveratrol and stigmasterol on the expression level of **(A)** anti-apoptotic protein Bcl-2, **(B)** FoxO3a, **(C)** catalase, **(D)** SIRT1, and **(E)** acetylated lysine (Ac-K). Representative of protein bands determined by Western blot with β-actin as loading control are presented at the upper panel. Quantification of protein expression level is presented as ratios of each protein/β-actin protein band intensity relative to the control group. Data are shown as the mean of three independent experiments ± S.E.M. (*p < 0.05 vs. control; ^#^*p* < 0.05 vs. H_2_O_2_-treated group).

In this study, the effects of stigmasterol on anti-apoptotic protein were determined using Western blot analysis. As shown in Figure 4A, the results revealed that treatment with 400 μM H_2_O_2_ reduced the expression of an anti-apoptotic protein Bcl-2 (70.7%). On the contrary, pretreatment with stigmasterol and resveratrol maintained Bcl-2 protein expression (103.7 ± 7.2% and 103.7 ± 5.4%, respectively) when treated with H_2_O_2_. These results indicated that stigmasterol and resveratrol prevented H_2_O_2_-induced apoptosis in the SH-SY5Y cells.

### Stigmasterol Effects on the Protein Expression of FoxO3a and Catalase

FoxO3a and catalase proteins were examined to study the role of stigmasterol in human neuronal cells under oxidative damage (Figures 4B,C, respectively). The protein levels of FoxO3a and catalase were suppressed when treated with H_2_O_2_ alone (67.8 ± 3.2% and 74.3 ± 0.9%, respectively). In contrast, FoxO3a (108.8 ± 5.8%) and catalase (107.7 ± 3.4%) were upregulated in the group pretreated with stigmasterol and were preserved under H_2_O_2_ induction. These results indicated that stigmasterol increased expression levels of both FoxO3a and catalase, similar to the group pretreated with resveratrol.

### Stigmasterol Effects on SIRT1 and Acetylated Lysine Protein Expression

Western blot assay was conducted to investigate SIRT1 and acetylated lysine proteins in stigmasterol-pretreated cells. It was revealed that the presence of H_2_O_2_ decreased SIRT1 expression (67.8 ± 2.9%). On the other hand, pretreatment with stigmasterol and resveratrol maintained SIRT1 protein levels compared with H_2_O_2_ exposure (Figure 4D). To determine the activity of SIRT1, the expression levels of acetylated lysine of the SIRT1 protein target were measured in this work (Figure 4E). Our data revealed that in contrast with SIRT1 expression levels, the levels of acetylated lysine were increased in the group treated only with H_2_O_2_ (127.3 ± 3.0%). While in the pretreatment groups, acetylated lysine levels were lower. These results indicated that stigmasterol increased SIRT1 expression and activated SIRT1-deacetylation reaction on its target protein consistent with resveratrol.

### Molecular Docking of SIRT1 and Stigmasterol

**Figure 5:**
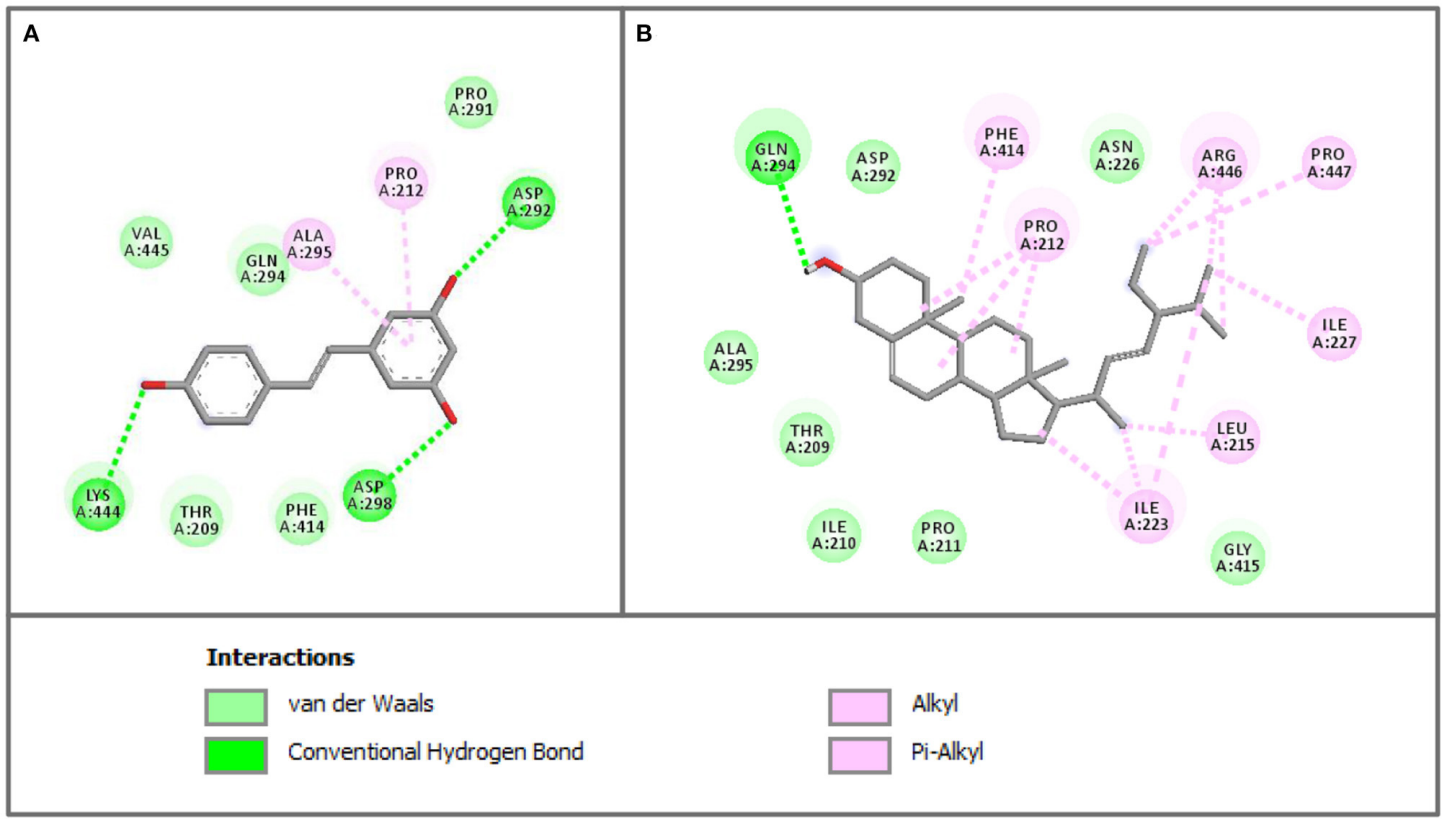
The 2D interaction profiles of resveratrol **(A)** and stigmasterol **(B)** in SIRT1 activator binding site (PDB ID 5BTR). The 2D structures of the compounds are shown *via* the gray line, interacting residues are represented as colored circles according to the type of interactions (2D diagram generated by Discovery Studio 4.5).

The crystallographic structure of human SIRT1 in complex with resveratrol was obtained from the Protein Data Bank using accession code 5BTR. Before performing the molecular docking procedures on the sets of compounds, the docking protocol was first validated for its robustness. This was performed by elucidating the structural difference of the co-crystalized ligand from that of the re-docked ligand by computing the root-mean-square deviation (RMSD). The protocol was considered acceptable as it afforded an RMSD value of ≤ 2.0 Å with a corresponding value of 1.138 Å. The calculated binding energy of stigmasterol (−8.1 kcal/mol) was comparable to that of the reference compound (resveratrol) (−8 kcal/mol). Binding interaction analysis of SIRT1 with stigmasterol and resveratrol revealed that both compounds bind to Thr209 (van der Waals interaction) and Pro 212 (hydrophobic interaction) in a similar fashion. Moreover, both compounds also bind to Phe414, Asp292, Gln294, and Ala295, as presented in Figure 5.

## Discussion

Phytosterols play an increasingly important role in the prevention of neurodegenerative disorders. Owing to their distinct BBB penetration properties, considerable attention has been given to the exploration of their neuroprotective effects. This study investigated whether pretreatment with stigmasterol displays protective effects against hydrogen peroxide-induced toxicity and examined molecular mechanisms in which stigmasterol exerts its neuroprotective effects in human neuronal cells. The imbalance between the production of ROS and antioxidants can cause oxidative stress. This phenomenon was observed in this study. Treatment with H_2_O_2_ leads to oxidative stress and cell death, as displayed by the increase of intracellular ROS leading to reduction of MMP and eventually significant loss of viable cells (52). One of the key events that lead to the reduction of MMP and eventually apoptosis is mitochondrial outer membrane permeabilization regulated by the Bcl-2 protein family including anti-apoptotic protein (Bcl-2) (53).

It was found that stigmasterol protected SH-SY5Y cells from oxidative stress-induced cell death, as measured from the increase of MMP and cell viability by reducing ROS levels, resulting in the decrease of cell apoptosis. Stigmasterol diminished intracellular ROS production and conserved cell intact and cell growth, thereby protecting the cells from apoptosis as indicated by the increase of anti-apoptotic protein (Bcl-2). This result is consistent with the previous study in which Bcl-2 inhibits apoptosis through regulation of cellular redox status, and acts directly as an antioxidant defense mechanism (54).

It was also observed that stigmasterol maintained SIRT1 protein expression against oxidative damage. Moreover, FoxO3a and catalase proteins were also found to increase thereby implying the antioxidative property of investigated compounds. In agreement with this observation, previous studies reported that SIRT1 activation could protect cells against oxidative stress by increasing catalase activity (55). Furthermore, SIRT1 deacetylases and activated FoxOs (i.e., particularly FoxO3a) subsequently stimulate the production of antioxidants (e.g., catalase and SOD) thereby promoting the resistance mechanism against oxidative stress (56, 57). Low levels of intracellular ROS and the high expression of catalase in the pretreated group indicated that catalase acted as an antioxidant defense mechanism by converting hydrogen peroxide into water (58). The role of catalase is prominent at higher levels of hydrogen peroxide (59).

This study also evaluated whether stigmasterol can activate SIRT1 by detecting the acetylated lysine protein levels following the incubation period. Interestingly, it was revealed that the levels of acetylated lysine were decreased in the cells treated with stigmasterol, indicating that SIRT1 was activated and subsequently stimulating a deacetylation reaction. The levels of acetylated lysine of the H_2_O_2_-treated group (127.3 ± 3.0%) were high and significantly different compared with the level of untreated and pretreated groups. Although which protein deacetylated by SIRT1 was not defined in this study, this result is the first indication that stigmasterol potentially activates SIRT1.

Owing to their prominent roles in cellular regulation and several disease progressions, research has been directed toward investigating the modulation of the sirtuin family by means of activating or inhibiting. Studies have shown that SIRT1 increases the production of α-secretase *via* deacetylation and activation of the retinoic acid receptor-β protein, which stimulates the transcription of the ADAM10 gene. The increases in ADAM10 drive the α-secretase cleavage of APP within the amyloid peptide region, resulting in the reduction of the Aβ peptide which gives rise to the characteristic amyloid plaques found in AD (10). *In vitro* activation of SIRT1 by either NAD^+^ or small molecule resveratrol has been shown to reduce the formation of Aβ oligomer by promoting APP metabolism by α-secretase (35). Recently, it has been found that in AD patients and elderly controls, the SIRT1 expression level was significantly reduced when compared with the healthy young controls (60). On the other hand, the clinical relevance of sirtuin inhibitor has also been reported. Among the most tested SIRT1 inhibitors, EX-527 has reached clinical trials for Huntington's disease, cancer treatment, and other pathologies [reviewed in (61)]. *In vivo* studies have proposed several pathways of SIRT1 inhibition by EX-527, including limiting cell proliferation stimulated by SIRT1 in a mice model of endometrial cancer, and increasing the rate of clearance of mHtt by increasing the acetylation of mHtt exon 1 in a mice model for Huntington's disease.

**Figure 6:**
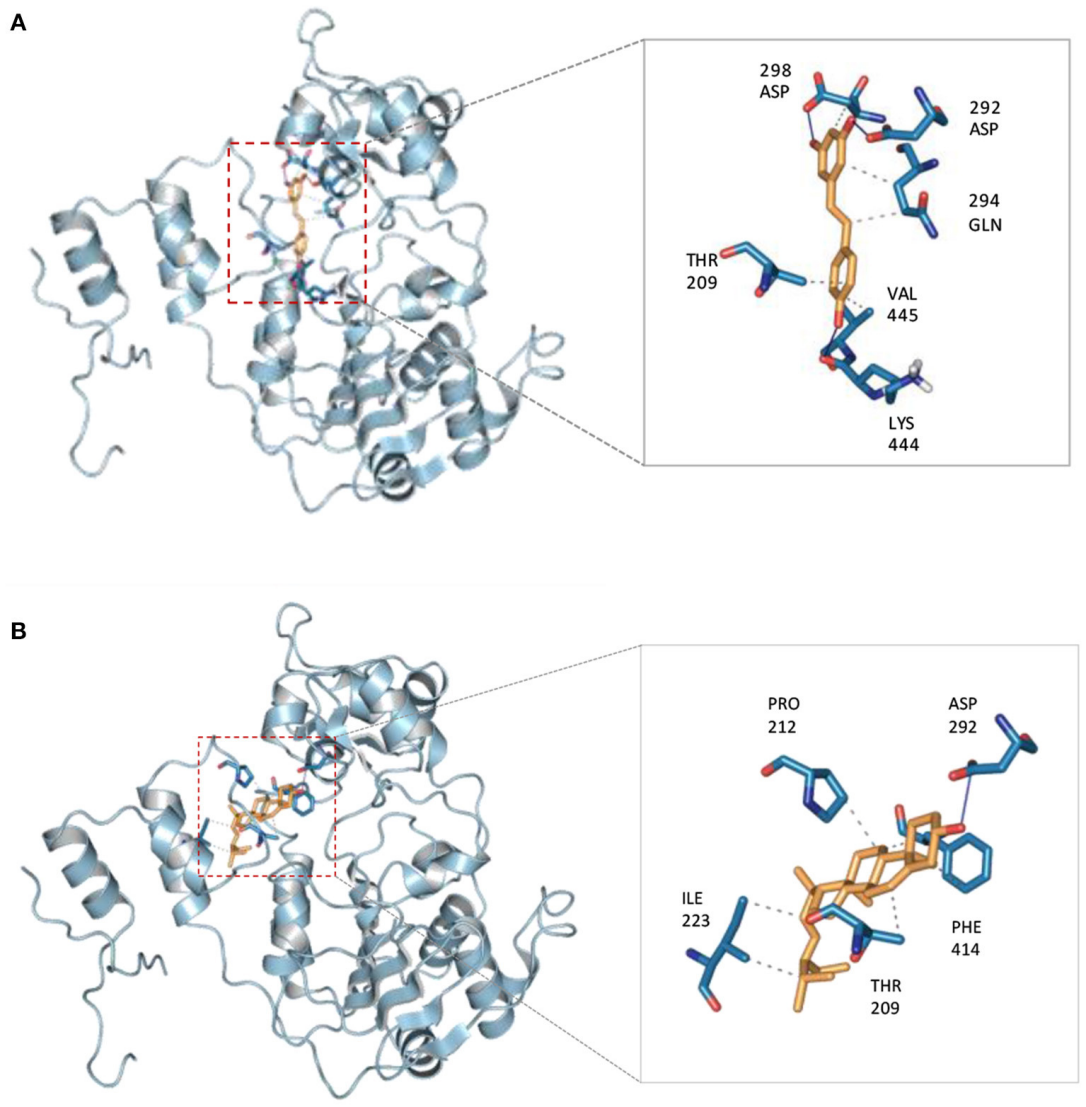
3D views of binding interaction of SIRT1 with **(A)** resveratrol compared with **(B)** stigmasterol.

The possible binding interaction between stigmasterol and the activator binding site of SIRT1 was investigated using the molecular docking method. The crystal structure of SIRT1 in complex with 3 molecules of resveratrol and AMC peptide was employed for molecular docking. Using the same binding site with resveratrol, it was revealed that the binding affinity of stigmasterol was similar to the resveratrol, and some of their binding residues were comparable. According to the binding analysis of SIRT1 with stigmasterol and resveratrol, both compounds bound in the same way to Thr209 with van der Waals interaction and to Pro 212 with hydrophobic interaction. In addition, both compounds also shared binding interaction with Phe414, Asp292, Gln294, and Ala295, as presented in Figures 5, 6. It was revealed that the binding interaction of SIRT1 and stigmasterol was also similar to other reported interactions of SIRT1 and activators, CWR peptide (62) and small molecule compound **1** (PDB ligand ID: 4TO) (63). Both of the reported SIRT1 activators and stigmasterol shared common binding residues with SIRT1 including Thr209, Pro212, and Asn226. In addition to these amino acid residues, stigmasterol and CWR peptide also bound to Phe414, Gly415, and Arg446 of SIRT1; whereas, stigmasterol and compound **1** similarly bound to Thr209, Pro212, and Asn226 of the activator binding domain of SIRT1.

Cao et al. (44) demonstrated that mutagenesis of N226A reduced SIRT1 activity rates, thus confirming the importance of the second resveratrol molecule binding with N226 residue. On the other hand, the mutation of D292A displayed a mild effect of SIRT1 activity reduction compared with the N226A mutation indicating that the third molecule of resveratrol was supportive. In addition, the mutation of R446A significantly decreased SIRT1 activity following the addition of resveratrol, suggesting that R446 is needed for internal domain arrangement. Therefore, the data indicated that the effect of the stigmasterol compound is comparable to the three resveratrol molecules to form a tight arrangement inside the binding domain.

## Conclusion

**Figure 7:**
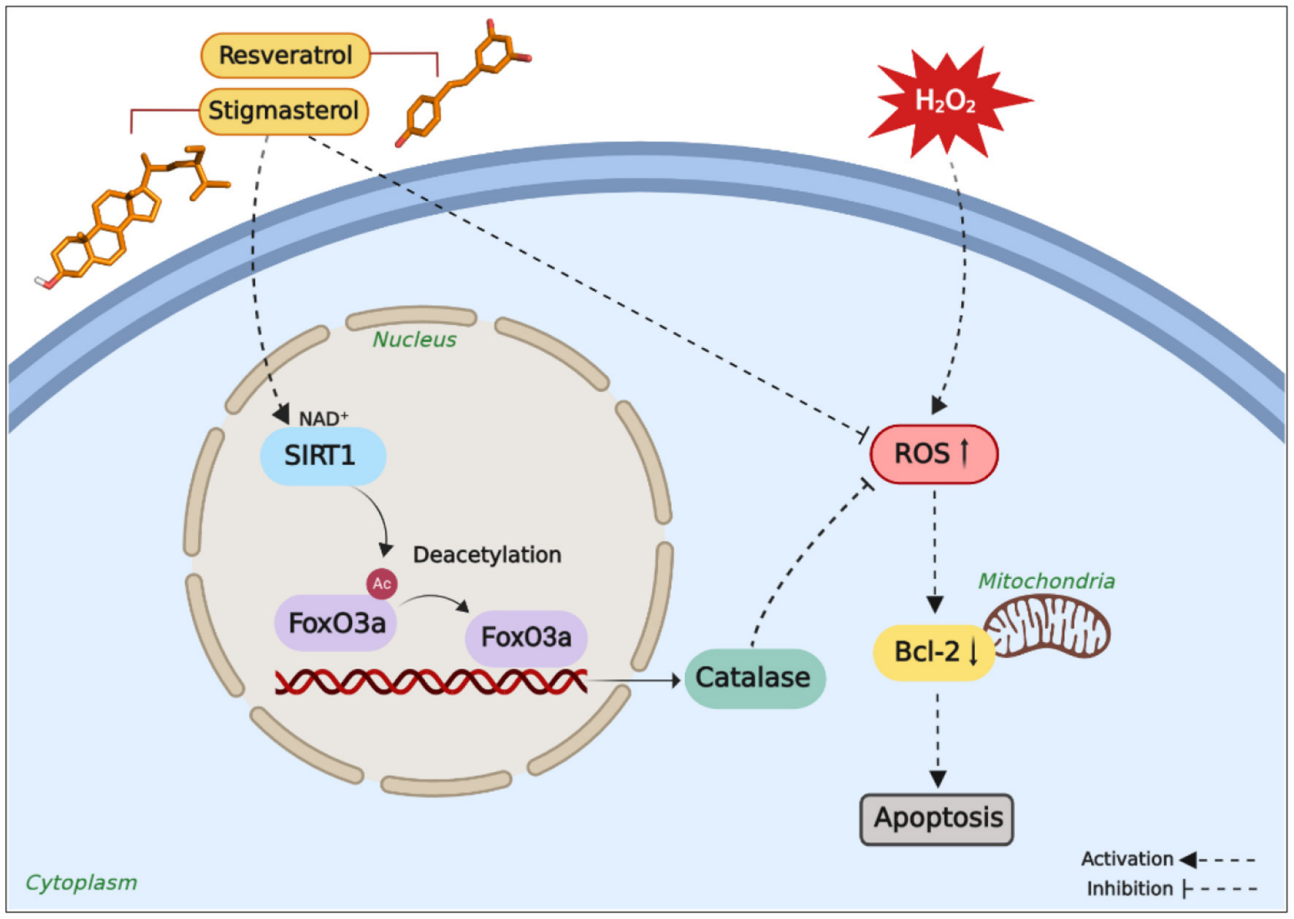
Potential neuroprotective pathways regulated by stigmasterol are comparable to resveratrol. Stigmasterol protects SH-SY5Y cells from H_2_O_2_-induced oxidative stress *via* stimulation of SIRT1-FoxO3a signaling pathway and promoting equilibrium of the antioxidant system, thereby reducing excessive ROS accumulation, attenuating neurodegeneration and apoptosis, and eventually promoting cell survival (Figure created with BioRender.com).

The results of the present study reveal the neuroprotective effects of stigmasterol against oxidative stress-induced apoptosis *via* the antioxidative defense mechanism of hydrogen peroxide reduction (Figure 7). Additionally, stigmasterol is also involved in attenuation against oxidative stress *via* SIRT1-FoxO3a modulation in the neurons. All in all, these findings suggest that this plant sterol is a promising bioactive compound for the prevention of neurodegenerative disorders. However, further study is still needed to better understand the underlying mechanisms in which stigmasterol exerts its neuroprotective effects.

## Data Availability Statement

The original contributions presented in the study are included in the article/supplementary material, further inquiries can be directed to the corresponding author.

## Author Contributions

RP, KP, and WR conducted the investigation and the methodology. RP and KP undertook formal analysis. WR contributed resources. RP, CN, and KP undertook visualization and wrote the original draft. CN, KP, WS, SP, and VP undertook writing, review, and editing. KP, WS, VP, and SP contributed to funding acquisition. All authors contributed to the article and approved the submitted version.

## Conflict of Interest

The authors declare that the research was conducted in the absence of any commercial or financial relationships that could be construed as a potential conflict of interest.
